# Collective Computation in Animal Fission-Fusion Dynamics

**DOI:** 10.3389/frobt.2020.00090

**Published:** 2020-07-21

**Authors:** Gabriel Ramos-Fernandez, Sandra E. Smith Aguilar, David C. Krakauer, Jessica C. Flack

**Affiliations:** ^1^Departamento de Modelación Matemática de Sistemas Sociales, Instituto de Investigaciones en Matemáticas Aplicadas y en Sistemas, Universidad Nacional Autónoma de México, Ciudad de México, Mexico; ^2^Unidad Profesional Interdisciplinaria en Ingeniería y Tecnologías Avanzadas, Instituto Politécnico Nacional, Ciudad de México, Mexico; ^3^Conservación Biológica y Desarrollo Social A.C., Ciudad de México, Mexico; ^4^Santa Fe Institute, Santa Fe, NM, United States

**Keywords:** social systems, distributed computing, inductive game theory, social information, animal foraging, collective intelligence

## Abstract

Recent work suggests that collective computation of social structure can minimize uncertainty about the social and physical environment, facilitating adaptation. We explore these ideas by studying how fission-fusion social structure arises in spider monkey (*Ateles geoffroyi*) groups, exploring whether monkeys use social knowledge to collectively compute subgroup size distributions adaptive for foraging in variable environments. We assess whether individual decisions to stay in or leave subgroups are conditioned on strategies based on the presence or absence of others. We search for this evidence in a time series of subgroup membership. We find that individuals have multiple strategies, suggesting that the social knowledge of different individuals is important. These stay-leave strategies provide microscopic inputs to a stochastic model of collective computation encoded in a family of circuits. Each circuit represents an hypothesis for how collectives combine strategies to make decisions, and how these produce various subgroup size distributions. By running these circuits forward in simulation we generate new subgroup size distributions and measure how well they match food abundance in the environment using transfer entropies. We find that spider monkeys decide to stay or go using information from multiple individuals and that they can collectively compute a distribution of subgroup size that makes efficient use of ephemeral sources of nutrition. We are able to artificially tune circuits with subgroup size distributions that are a better fit to the environment than the observed. This suggests that a combination of measurement error, constraint, and adaptive lag are diminishing the power of collective computation in this system. These results are relevant for a more general understanding of the emergence of ordered states in multi-scale social systems with adaptive properties–both natural and engineered.

## 1. Introduction

In an influential framework for studying animal social organization, Hinde (1976) stressed that both animal and human societies are multiscale. Short-term interactions between pairs of individuals lead to longer-term social relationships and social structures, with social relationships arising as individuals generalize from a history of social interactions. Hinde noted that individuals classify social relationships into types (kin, matriline, etc.) regardless of the individuals involved. The idea that primates use abstraction to make sense of their world has been shown in a number of studies subsequent to Hinde (1976) (e.g., Cheney and Seyfarth, 1990, 2008).

Over a series of papers, Flack et al. (Flack, 2012, 2017a,b; Flack et al., 2013; Daniels et al., 2017; Brush et al., 2018) have been developing a theory of collective computation (inspired in part by Hopfield's collective computation in neural networks Hopfield, 1982, 1984; Tank and Hopfield, 1988). In the context of animal behavior, this work links Hinde's (1976) generalization and abstraction processes to the formation of collectives. In Flack and Krakauer's formulation, components (for the purposes of this paper, individuals) reduce uncertainty about the environment or state of a system by coarse-graining fast microscopic behavior (Flack, 2017a). An example of uncertainty reduction would be over the cost of social interaction (Flack, 2012). When coarse-grainings converge (meaning the estimates of regularities are largely shared by individuals), this can produce a coherent mesoscale (e.g., a social network or circuit). This can then function like an information bottleneck (Tishby et al., 2000; Tishby and Zaslavsky, 2015; Flack, 2017a): the strategies, as coarse-grainings, capture regularities individuals perceive in the physical or social environment. The way individuals combine strategies to make decisions in the collective captures the regularities they perceive as most important. Emergent from these slowly changing mesoscopic individual strategies and collective metastrategies is social structure. As a social structure consolidates and individuals start to “reference it” for decision-making, it feeds back through effective downward causation (Flack, 2017a) to modulate the cost of social interaction or interaction with the environment. Once complete, this process can give rise to a new scale, and under suitable conditions, novel functions.

To make this concrete, consider as an example the collective computation of power structure in macaque societies (reviewed in Flack, 2012, 2017a). Individuals summarize fight histories using unidirectional signals. The sender emits the signal once it perceives it is likely to loose a fight. The signal reduces uncertainty in the receiver that the sender agrees to subordination—willingness to yield in future interactions. Encoded in the consolidating network or circuit of signals between group members is information about the distribution of power. Hence the power structure is computed as individuals estimate regularities about fighting abilities and share these opinions with the receiver and other group members via signals. Through this process, different levels of organization arise at successively slower timescales: fights (fast), signaling (slow), and power structure (slowest). The process of generating coarse-grained, slow variables (the signals, properties of the circuits) is the outcome of individual strategic computations (interaction and signaling decisions) that aggregate into an output collectively estimated to fit the state of the environment (Flack, 2017a,b). This two-part process of information accumulation and aggregation makes up collective computation (Daniels et al., 2017; Flack, 2017a).

Among other examples in the animal behavior literature that might result from collective computation are coordinated foraging and predator avoidance in animal groups (Couzin et al., 2003; Gordon, 2016; Sosna et al., 2019), rapid direction changes during collective motion in fish schools and bird flocks (Hein et al., 2015), and distributed foraging in social insects (Gordon, 2016).

Fission-fusion social dynamics, in which individuals fission and fuse into subgroups of varying size, is a collective pattern arising from individual decisions (Sueur et al., 2011; Ramos-Fernández et al., 2018). These dynamics are thought to be adaptive, as they allow individuals to forage more efficiently in heterogeneous environments, share information about the location of resources, and adjust the size of their subgroups to resource availability (Aureli et al., 2008; Sueur et al., 2011; Palacios-Romo et al., 2019). The individual, strategic decisions to leave or join subgroups, how these decisions influence subgroup size distributions, and whether these are a good fit or even predicted by environmental states, are open questions. Previous work on spider monkeys suggests individuals change their strategies based on environmental states to include the rate at which they encounter fruit and the presence of knowledgeable individuals in social networks (Ramos-Fernández and Morales, 2014; Palacios-Romo et al., 2019).

We study how individual spider monkeys use social knowledge (information accumulation) to collectively compute adaptive subgroup size distributions (information aggregation). We use inductive game theory (DeDeo et al., 2010; Krakauer et al., 2010) to extract stay-leave probabilistic strategies from a time series of subgroup composition. The strategies constitute the microscopic input to the collective computation. From the microscopic input we construct a family of circuits in which nodes correspond to individuals and edges, weighted by probabilities obtained from the data, specify probabilistic rules—strategies—for remaining in or leaving a subgroup. Circuits capture variation in the way individuals integrate over their strategies (see section 3) to decide to stay or go.

Each circuit serves as a mesoscopic hypothesis for how strategies combine to produce decisions and how decisions combine to compute subgroup size distributions. In a computational language, the inputs (individual strategies) combine to produce an output (a subgroup size distribution). We run the circuits forward in simulation to determine how individuals combine strategies and hence how many information sources they take into account to make decisions. We construct a food abundance index based on the size and abundance of fruiting trees and calculate the transfer entropy between this index and the distribution of subgroup size in order to determine whether the circuit that best recovers the observed subgroup size distribution is also optimally computing the state of the environment.

## 2. Data

**Figure 1:**
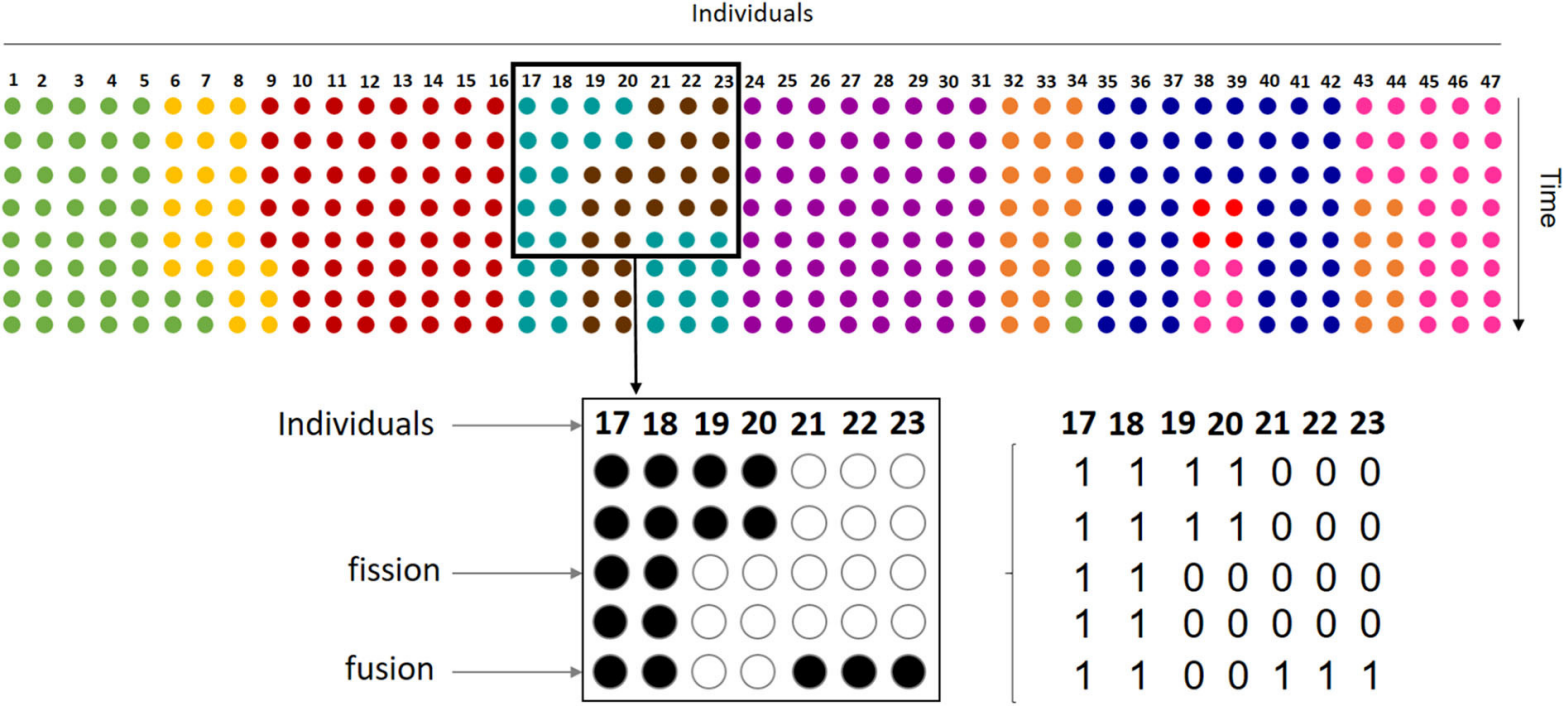
Our dataset samples the process of fission-fusion dynamics in the entire group. Each row with colored circles on the top of the figure represents how the 47 individuals that conform the spider monkey group are sorted into subgroups at a given moment, with each color indicating subgroup membership. Thus, in the first row or time step represented, the group is organized in 9 different subgroups and, throughout the remaining steps, subgroups change size and memberships by individuals leaving (fission) and joining (fusion). Our sample includes only one subgroup followed at any time, so we have information on the presence or absence of each group member on the observed subgroup. For example, the bottom part of the figure shows observations from one subgroup (turquoise dots on the top). Here, rows represent 5 instantaneous scan samples taken every 20' on individuals 17 thru 23, where each individual can be either present (full circles) or absent (empty circles). In this case, the subgroup shows a fission of two individuals in the third scan and the fusion of three at the fifth scan. For analysis, we coded data as binary vectors corresponding to each scan sample.

Subgroup composition data were collected in Punta Laguna, Yucatan, Mexico, as part of a long-term study of social behavior using identified individuals (details about study site and subjects can be found in the Supplementary Information). Data consist of scan samples of subgroup composition, taken every 20' during an average of 5 h. per day throughout 2 years (Jan. 2013–Dec. 2014), for a total of 5,780 scan samples. A total of 47 known adult, sub-adult and juvenile individuals were observed during this period (see Supplementary Table 1). Thus, each sample is a vector of 47 binary digits, with 0 corresponding to an absence of the individual in the *ith* position and 1 corresponding to a presence (Figure 1). Continuous series of scans, averaging 8.4 scan samples (± 3.9 SD), include uninterrupted follows of a subgroup in which at least one individual remained during the full series. Given that the typical duration of a subgroup is 1.5 h. (Pinacho-Guendulain and Ramos-Fernández, 2017), a subgroup may persist over multiple scans. The temporal resolution of this sampling regime was maintained in the analysis in order to obtain a sufficient number of continuous series of scans. Had we resampled the original dataset at a larger temporal scale, we would have lost an important number of continuous series. Also, the persistence of a subgroup over several scans implies that individuals in a subgroup are tolerating one another, which is informative about the weight of their mutual influence (see below).

The raw data supporting the conclusions of this manuscript will be made available by the authors, without undue reservation, to any qualified researcher.

## 3. Microscopic Strategy Extraction and Distribution

We distinguish between strategies and decisions. A decision is binary: to leave or stay in a subgroup (in the original inductive game theory work, to join or avoid a fight, DeDeo et al., 2010). Strategies (called Δ*P*, as in previous work, DeDeo et al., 2010) are “above-null” probabilities (see below for calculation) describing the weight of individual A's presence or absence in the *current* subgroup (as determined by scan sampling, see section 2) on individual B's decision to stay or go from the subgroup in the subsequent sample. Here and in previous work (DeDeo et al., 2010), multiple individuals can influence individual B. Hence B will have multiple strategies and, in the limit, a strategy for every other group member. We address how B integrates strategies to reach a decision in section 4. Here we quantitatively describe how we define and extract strategies from the time series. We end up with a list of pair-wise strategies for which our extraction method indicates above-null support in the time series. We do not consider higher order strategies as in DeDeo et al. (2010).

For all pairs of individuals {A:B, A:C, A:D,.}, we calculate the probability an individual B is present or absent in a sample if individual A was present in the previous sample within the same continuous series of scans:

(1) $$\begin{array}{l} \;P\left(A\to B\right)=\frac{N\left(B_{t+1}|A_{t}\right)}{N\left(A\right)}, \end{array}$$

where *N*(*B*_*t*+1_∣*A*_*t*_) is the total number of times B was present at time *t*+1 given that A was present at time *t* within a continuous series of scans and *N*(*A*) is the number of times A was present in all samples.

As with previous work (DeDeo et al., 2010), to remove time-independent effects from the transition probabilities (for example, due to general differences in gregariousness), we calculate the difference between the probability inferred from the data and a null expectation:

(2) $$\begin{array}{l} \Delta P\left(A\to B\right)=\frac{N\left(B_{t+1}|A_{t}\right)-N_{null}\left(B_{t+1}|A_{t}\right)}{N\left(A\right)}, \end{array}$$

where *N*_*null*_(*B*_*t*+1_∣*A*_*t*_) is the average number of times B is present at time *t*+1 given that A is present at time *t* within a continuous series of scans, calculated from 1,000 bootstrapped permutations of the data.

Similarly, we consider the weight of A's absence on the presence of another individual B in a subsequent sample:

(3) $$\begin{array}{l} \;P\left(!A\to B\right)=\frac{N\left(B_{t+1}|!A_{t}\right)}{N\left(!A\right)}, \end{array}$$

and

(4) $$\begin{array}{l} \Delta P\left(!A\to B\right)=\frac{N\left(B_{t+1}|!A_{t}\right)-N_{null}\left(B_{t+1}|!A_{t}\right)}{N\left(!A\right)}, \end{array}$$

where *N*(*B*_*t*+1_∣!*A*_*t*_) is the number of times B is present in a sample when A is absent in the previous sample within a continuous series of scans, *N*(!*A*) is the number of times A is absent in all samples, and *N*_*null*_(*B*_*t*+1_∣!*A*_*t*_) is the average of the same number for 1,000 bootstrapped versions of the original data.

These Δ*P* constitute the pair-wise weight of each group member on a given individual's binary decision to leave or join a subgroup.

**Figure 2:**
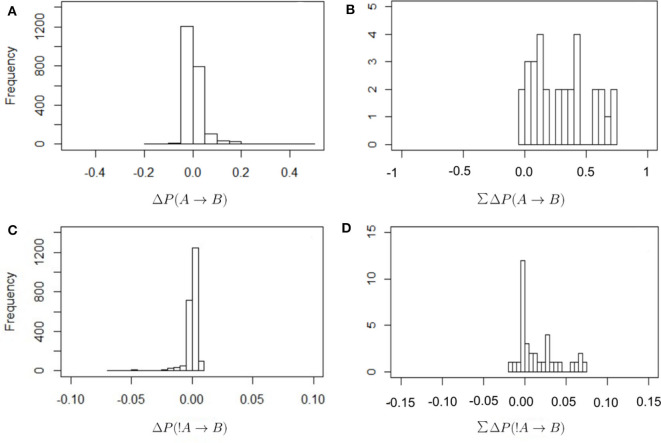
Frequency distribution of the values of Δ*P* for the different combinations of dyadic weights, as defined in Equations (2) and (4) **(A,B)** and for the total sum of the incoming weights that each individual receives (**C,D**; this is the in-strength of nodes in Figure 3). The values of Δ*P*(*A* → *B*) have a wider distribution around zero, with correspondingly higher total values of in-strength, than in the case of Δ*P*(!*A* → *B*).

Figure 2 shows the frequency distribution of the values of Δ*P* as defined in Equations (2) and (4). In all cases values are centered around zero, with the values of Δ*P*(!*A* → *B*) closer to zero than in other cases. This is because the denominator in Equation (4) is larger than in Equation (2), as it includes all instances of individual A being absent from the observed scan. There are proportionally fewer cases in which B is present after an absence of A because there are many cases where A is absent. Thus, these values of Δ*P*(!*A* → *B*) should be interpreted with care. It is also the case that most values of the total sum of weights received are positive. In other words, most individuals receive a total positive weight from the presence or absence of strategically connected individuals. Only a few cases show a total negative weight of the presence or absence of others.

We identified significantly positive dyadic weights as values of Δ*P* higher than the 95% percentile of the permuted values for each dyad. Accordingly, significantly negative dyadic weights were values of Δ*P* lower than the 5% percentile of the permuted values for each dyad.

## 4. Mesoscopic Circuit Construction

**Figure 3:**
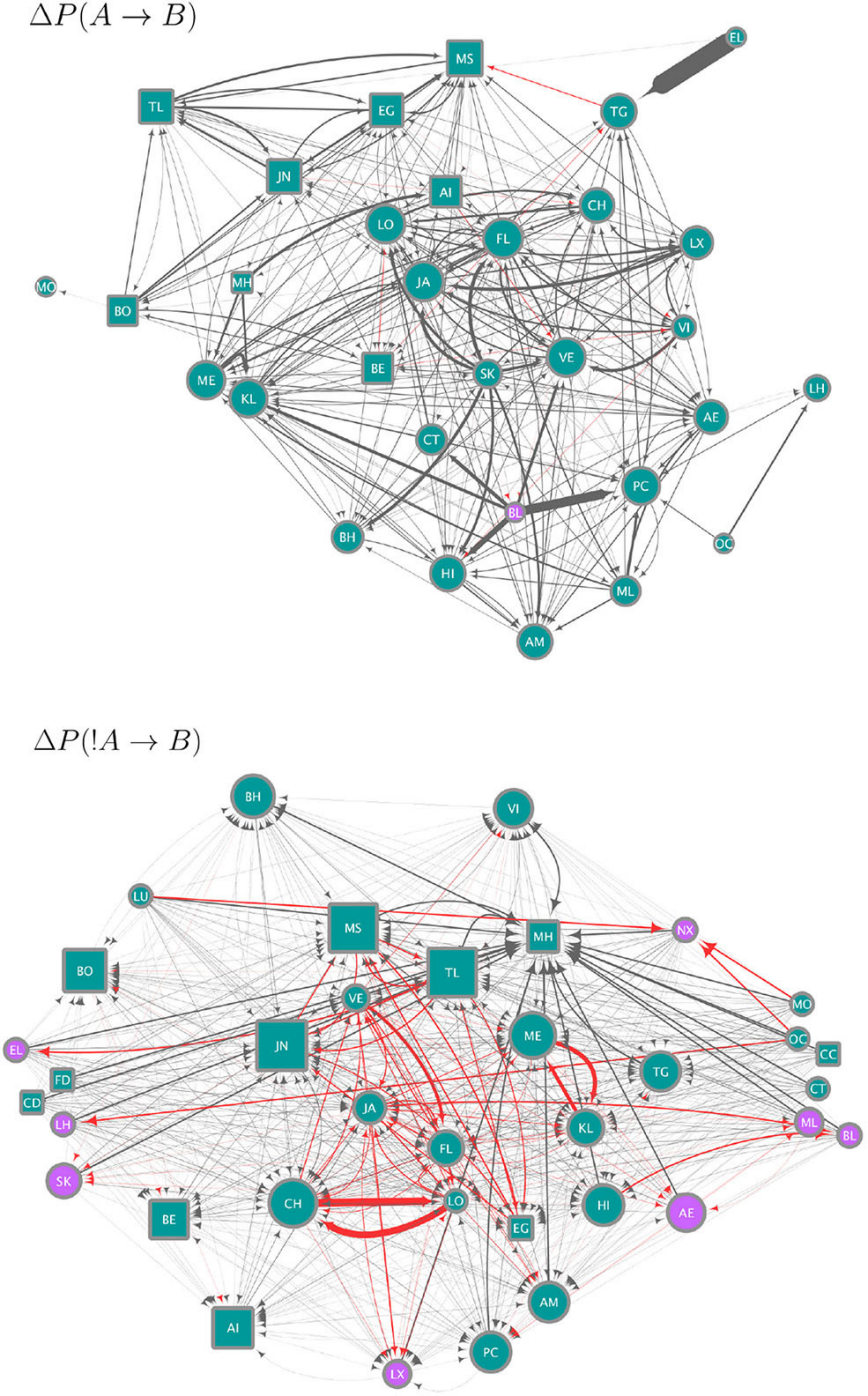
Circuits showing the strategies (significant, pairwise negative and positive weights) extracted from the data and as defined in Equations (2) (upper panel, Δ*P*(*A* → *B*)) and (4) (lower panel, Δ*P*(!*A* → *B*)). Nodes correspond to individuals indicated by two-letter codes and their shape represents females (circles) and males (squares). Only for the purposes of this visualization we removed the 11 juvenile individuals, who do not move independently of their mothers. However, they were included in the analyses of Δ*P* values. Edges correspond to significant Δ*P* values, of a width proportional to their value. Each circuit employs a different range of Δ*P* values, as Δ*P*(*A* → *B*) values range from −0.00076 to 0.3 and Δ*P*(!*A* → *B*)) values from −0.00033 to 0.00315 (see Figure 2). Node size is proportional to the in-strength of the node, i.e., the total significant weight from others as defined by the sum of the incoming Δ*P* values. Node color corresponds to whether the node has a positive (blue) or negative (purple) in-strength. The color of edges corresponds to negative (red) and positive (gray) values.

We use the strategies obtained from the data to construct circuits (*i.e*. the set of all significant Δ*P* values as weights between all pairs of individuals; this is the mesoscopic level of our analysis) each of which is a hypothesis for (1) how individuals integrate over their strategies to arrive at a binary decision to join or leave a subgroup and, (2) specify how the resulting decisions combine to produce the distribution of subgroup size. The circuits in Figure 3 give a qualitative summary of significant strategies. For each individual, there are 46 potential weights (significant Δ*P* values) from either the presence or absence of others at scan time *t*, which could determine its presence or absence at scan time *t* + 1. The circuits in Figure 3 show only 31 individual nodes for Δ*P*(*A* → *B*) and 36 for Δ*P*(!*A* → *B*), who were involved in significant weights. On average, each individual in these circuits is linked to 20.25 (± 1.98 SE) other individuals in the Δ*P*(*A* → *B*) and to 31.67 (± 1.40 SE) in the Δ*P*(!*A* → *B*) circuit (Figure 3). Similarly, whereas each of the circuits in Figure 3 could have up to 1,260 links, the Δ*P*(*A* → *B*) circuit has 314 and the Δ*P*(!*A* → *B*) circuit 570 links. Supplementary Figure 1 shows the values of all significant weights included in these circuits, as well as the individual instrength and outstrength.

The circuit for Δ*P*(*A* → *B*) (upper panel in Figure 3) represents significant weights of the presence of individual A at scan *t* on the presence of individual B at scan *t*+1. Most of the values of Δ*P*(*A* → *B*) were positive or close to zero (see Figure 2A), therefore this circuit contains mostly positive weights (gray links), corresponding to weights of attraction. There is an apparent homophily by sex in this circuit, with individuals influencing other individuals of the same sex more than those of the other. Other attractive interactions are those between some pairs of adult females and their subadult daughters (e.g., females VE-VI and JA-LX in the upper panel of Figure 3, CH-LO and ME-KL in the lower panel). Individuals differ in their in-strength values (as can be observed in Figure 2B) with the individuals with the highest values of in-strength receiving many different weights, some with high values of Δ*P*, both females and males. Only one individual (female BL) had a negative in-strength value, implying that it received a total negative Δ*P*(*A* → *B*) higher than the total positive Δ*P*(*A* → *B*).

The circuit for Δ*P*(!*A* → *B*) shows a different picture (lower panel in Figure 3). Here values were skewed below zero, although overall they were much closer to zero than the values of Δ*P*(*A* → *B*) (Figure 2). Even considering that the variation around zero is small, this circuit contains both positive and negative weights, corresponding to repulsion and attraction, respectively, but the most important links are negative or attractive. There is, as in the previous circuit, evidence of some degree of homophily, with individuals of the same sex influencing each other through negative links more than those of the opposite sex. Conversely, a high proportion of positive or repulsive links occur between the sexes. Both males and females have high values of in-strength, although those with a negative in-strength (receiving many negative, attractive weights) in this circuit were all females. Individuals with the highest values of positive in-strength (corresponding to a total sum of positive or repulsive weights in this network) were males.

Each individual can have multiple strategies, and they can be in conflict (DeDeo et al., 2010), with some weights positive and others negative. In addition, the weight or importance (given by Δ*P*) of each strategy varies. Hence individuals must integrate over their set of strategies to make a decision about whether to join or leave the subgroup. Figures 2B,D show frequency histograms for these incoming values, corresponding to the in-strength of the nodes in Figure 3. These in-strength values can be understood as the likelihood that an individual will be influenced by others: an individual with a high in-strength is more likely to decide to be present due to another individual's presence (in the case of Δ*P*(*A* → *B*) values, upper panel in Figure 3) or absence (in the case of Δ*P*(!*A* → *B*) values, lower panel in Figure 3) than another individual with a lower in-strength.

**Figure 4:**
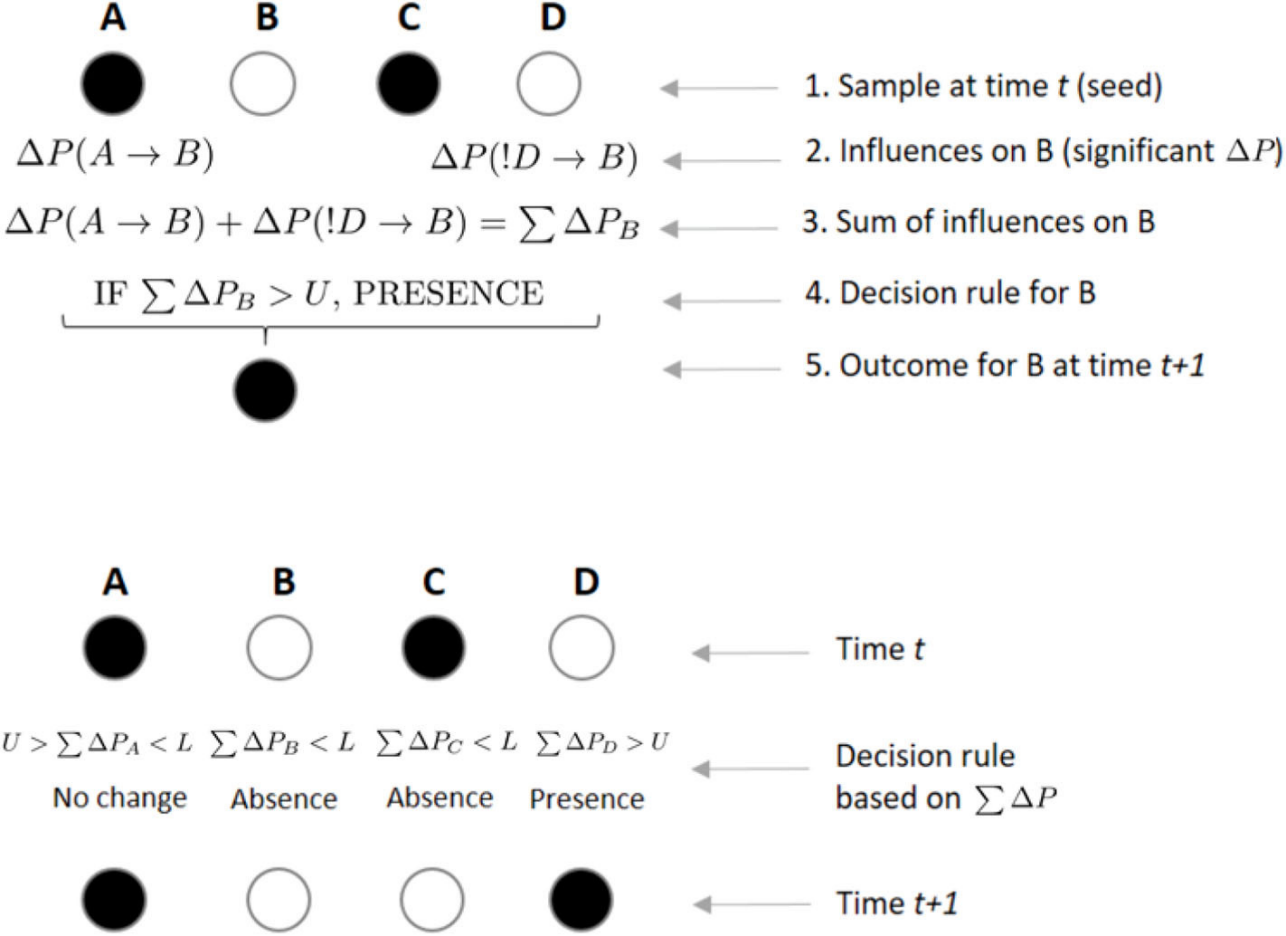
Example of rules by which individuals integrate incoming weights to decide their state at scan *t* + 1. In the upper panel, an individual B integrates incoming weights using a decision rule. If B, who is not present in the focal subgroup at scan *t*, receives a significant weight Δ*P* from the presence of A on its presence at scan *t* + 1, while receiving another significant weight from individual D's absence on its presence, B will integrate both weights by a simple sum. If this sum ∑Δ*P* is above a certain threshold *U*, B will decide to join a subgroup where it was not present at time *t*. In the lower panel, each individual arrives to its own value of ∑Δ*P*, which will determine its presence or absence from the subgroup at time *t* + 1, depending on the values of *U* and *L*. A sum of Δ*P*s greater than *U* or smaller than *L* could lead an individual to either maintain (e.g., B was absent in time *t* and its ∑Δ*P* is below *L*, leading to its absence in *t* + 1) or change its previous state (e.g., C was present in time *t* and ∑Δ*P* is below *L*, leading to its absence in *t* + 1).

We further assume that at any given time *t*, if the sum of significant Δ*P* values ∑Δ*P* directed toward an individual B is positive and greater than a threshold *U*, B will be present on the sample at *t*+1 (irrespective of whether it was present or absent in the previous sample; Figure 4). Conversely, if ∑Δ*P* is negative and smaller than a threshold *L*, individual B will be absent from the following sample (again, independently of whether it was present or absent in the previous sample). However, if *L* < ∑Δ*P*<*U*, then there is no effect from others and B remains in the same state as in the previous sample (i.e., present if it was present at time *t*, absent if it was absent; Figure 4). Thus, *U* is a threshold parameter controlling how likely it is for individuals to be present in a subgroup based on the weight of others. The value *L* controls the opposite, i.e., how likely it is that individuals will be absent in a subgroup based on the weight of others. Note that the total sum ∑Δ*P* includes both the Δ*P*(*A* → *B*) and the Δ*P*(!*A* → *B*) values, such that an individual would be integrating the weights it receives across both circuits shown in Figure 3. At higher values of *U*, the presence of an individual in a subgroup is less likely to be influenced by others. In that sense, high values of *U* imply less interdependence of individuals in their decisions to be present or not in a subgroup. Conversely, *L* controls the opposite end of the range of values of ∑Δ*P*, such that at more negative values of L, an individual should be less likely to be absent from a subgroup due to the previous weight from others. We tested *U* = {0.0001, 0.001, 0.01, 0.1, 0.2, ..., 0.9} and *L* = {−0.9, −0.8, ..., −0.1, −0.01, ..., −0.00001}.

Different individuals could actually be using a different value of the *U* and *L* thresholds, or the values could change over time, depending on slower ecological variables such as the dry and wet seasons or even longer timescales related to the ecological succession of the forest in the spider monkey's habitat. In this work we assume, as a first approximation, a single value of the threshold parameters for all individuals and seasons.

There are also subtle points here concerning how strategies are aggregated by individuals to produce binary decisions. In previous work (DeDeo et al., 2010), higher order (triadic—C only joins current fight if both A and B were present in the previous fight) as well as pair-wise strategies (A joins if B was previously present) were extracted from time series data and a circuit was constructed for each strategy class. Preliminary analyses in that work suggest these triadic strategies are non-decomposible into two pair-wise strategies (i.e., not reducible to additive individual or pair-wise interactions; Daniels et al., 2016; Chen et al., 2019). Individuals typically had multiple higher-order strategies and so, as with pair-wise, higher-order strategies were pushed through gates to produce binary decisions. Here we allow for the possibility that individuals take into account multiple strategies and hence be under the influence of multiple individuals, but we do not explore whether the interactions are pair-wise or higher-order.

We use these circuits to generate, by simulation, new datasets from the original dataset. In what follows, we restricted our analyses and simulations to a subset of the original dataset that included the same months for which food abundance data was available (Sep. 2013–Sep. 2014; see section 6), corresponding to 3,032 scan samples. We started by randomly choosing a scan sample (subgroup) that serves as the “seed” or first scan of a sequence of *n* samples, where *n* is randomly drawn from the frequency distribution of the number of samples per continuous observation period in the original biweekly period. Thus, the seed establishes which of the 47 monkeys in the group are present or absent in the first sample. Because the seed and the duration of continuous observation periods are selected within observation periods, simulated data contain information about the variation in subgroup size and composition between bi-weekly periods. If an individual A is present in the first scan, the simulation looks at values of Δ*P*(*A* → *B*) and considers any significant values or weights of A on others. If, on the contrary, A is not in the seed, then the simulation looks for significant values of Δ*P*(!*A* → *B*). This applies to all 47 individuals.

These rules are used to determine subgroup composition of the *n* samples in the continuous observation period. This is repeated for 633 sequences, corresponding to the number of continuous observation periods in the original dataset. In total, we generated 100 simulated datasets for each combination of thresholds *U* and *L*.

## 5. Testing Circuits in Simulation

Here we assess how individuals integrate strategies to make decisions Δ*P* and how decisions combine to compute the subgroup size distribution. We do so by asking which circuit, given an integration threshold, produces a simulated data set with a distribution of subgroup size that best recovers the observed one. We used each set of 100 simulated datasets with different values of *U* to evaluate the set of subgroup size distributions that is in closest correspondence to the observed. We only show the effects of varying *U* at *L* = −0.00001, since the variation in *L* for any value of *U* does not have an effect on the subgroup size distribution. This is likely because values of ∑Δ*P* are mostly positive (Figures 2C,D), so very few values are below the *L* threshold. In other words, even the smallest negative value of *L* has no effect on the tendency of individuals to modify their presence based on the presence or absence of others.

**Figure 5:**
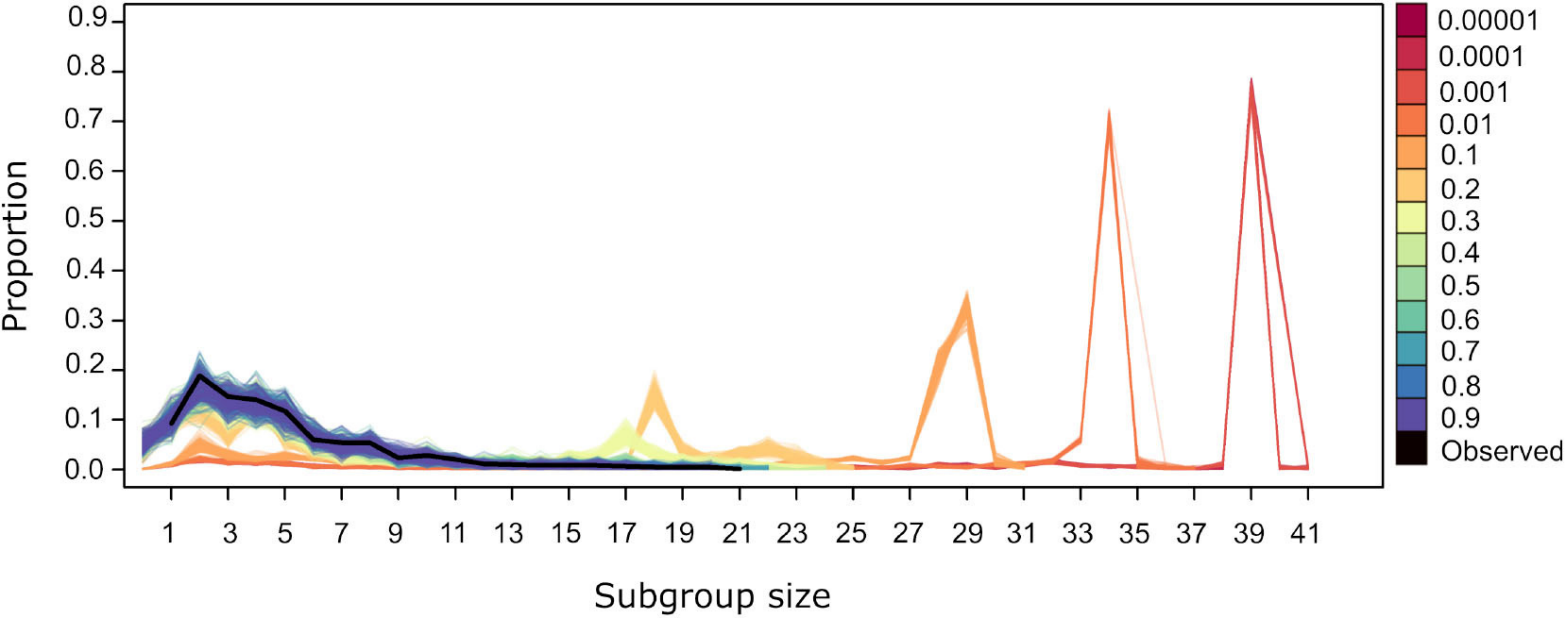
Subgroup size distribution for the original dataset (black thick line) and for the simulated datasets. Lines of a given color correspond to the resulting distribution from 100 repetitions using different values of *U*, with *L*= −0.00001.

For values of *U* = 0.4 and above the subgroup size distribution from simulated datasets is similar to the observed (Figure 5). Values of *U* <0.4 generate distributions where small subgroups are underrepresented and larger subgroups are overrepresented. This is due to the fact that, at lower values of *U*, individuals are more likely to be influenced by others, both through the significant values of Δ*P*(*A* → *B*) and Δ*P*(!*A* → *B*). The former dominate the dynamics of subgroup size change because they have higher and positive values overall (Figure 2). Thus, when *U* <0.4, individuals are aggregating more frequently, deciding to join subgroups at higher frequency as in the observed data. Values of *U* <0.4 give rise to subgroups converging at a single size for each value of *U* (Figure 5). This may be due to all individuals deciding to join subgroups, even those without significant weights, as must be the case in subgroups larger than 36, the number of nodes in the largest network in Figure 3 that depicts all individuals that are involved in significant weights.

**Figure 6:**
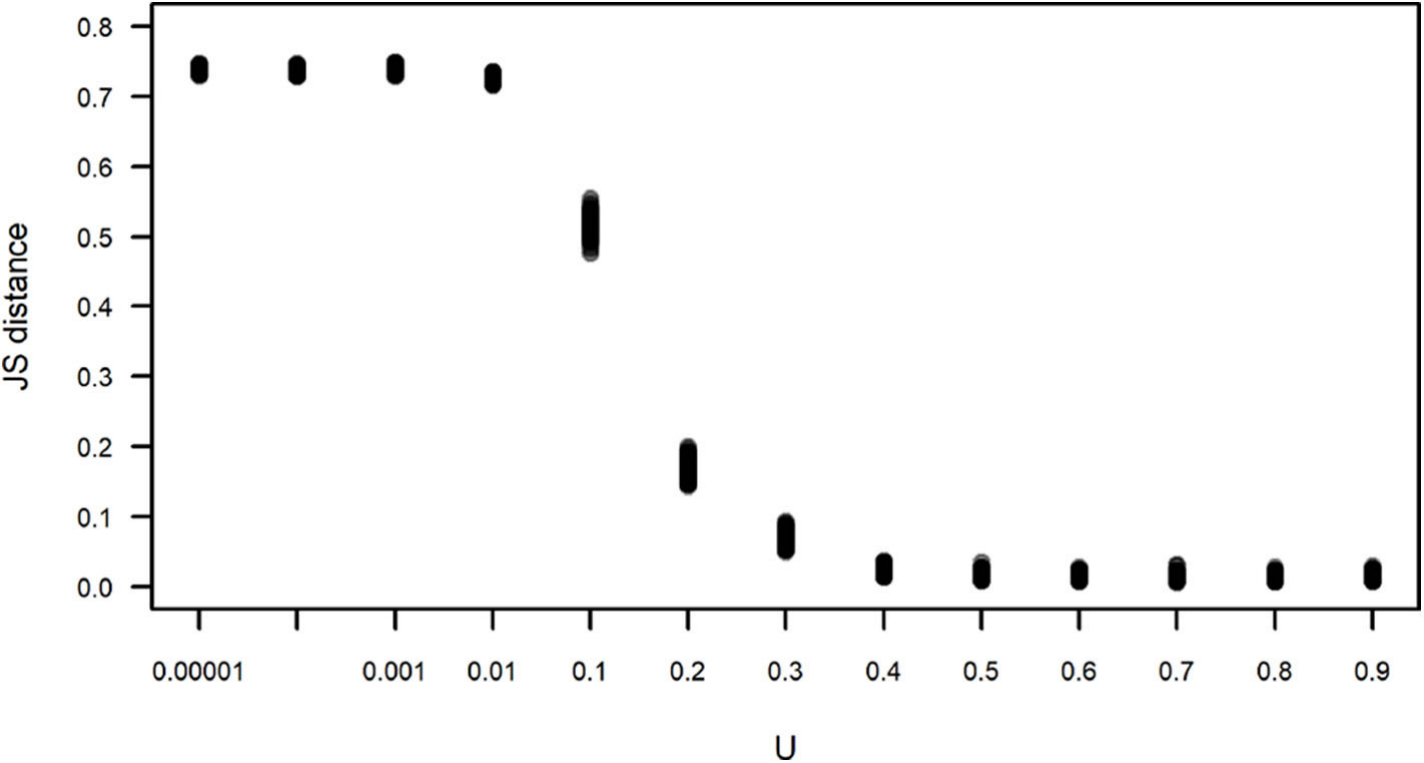
Jensen-Shannon (JS) distance between the observed and simulated subgroup size distributions shown in Figure 5. Each dot corresponds to the JS distance between an instance of 100 simulations for each value of *U*. For all simulations, *L* = −0.00001.

We compared the observed subgroup size distribution and those obtained by simulation under different values of *U* using the Jensen-Shannon distance (Figure 6). This distance between two random variables *x* and *y* is defined as:

(5) $$\begin{array}{l} JS\left(x|y\right)=H\left[\frac{x+y}{2}\right]-\frac{1}{2}\left[H\left(x\right)+H\left(y\right)\right] \end{array}$$

where *H* is the entropy of each variable, $p\left(x\right)\frac{1}{logp\left(x\right)}$ and *X* and *Y* are, in this case, the observed subgroup size and the subgroup size obtained in one run of a simulation, respectively. Figure 6 corroborates what is apparent in Figure 5, that simulations run with *U*≥0.4 yield subgroup size distributions that are closer and indistinguishable from the observed distribution, with *JS* values that are close to zero, while simulations run with *U* <0.4 have an increasing *JS* with respect to the observed. Simulations run with all values of *L* for *U*=0.4 yield subgroup size distributions that are equally close to the observed (data not shown).

## 6. Fit of Output to Environment

A central question is whether the collective computation output is adaptive (Flack, 2017a; Brush et al., 2018). Previous studies of spider monkeys suggest there is a weak relationship between subgroup size and food abundance (Symington, 1988; Pinacho-Guendulain and Ramos-Fernández, 2017). In general, subgroups tend to be larger during periods of high food abundance. This suggests that subgroup size can track the abundance of resources. Here, we investigate whether subgroup size distribution is predicted by the relative abundance of fruiting trees.

We use data from a 1-ha plot where all the trees (diameter at breast height, *D* > 10 cm) from the 15 most consumed species by the monkeys, were monitored bi-weekly for a year from September 2013 to September 2014, comprising 25 monitoring periods. A total of 487 trees were identified, their *D* was recorded, and every 2 weeks they were assessed for the presence of fruit. The data obtained were used to calculate the proportion of trees with fruit available in a given period expressed in terms of the total tree *D* rather than tree number. To do so we calculated the sum of the *D* values of all the trees with fruit (*D*_*f*_) in period *p* divided by the sum of *D* values for all the trees in the plot (*D*_*i*_), giving an index of food abundance for a period *p*, *IFA*_*p*_ = ∑*D*_*f*_/∑*D*_*i*_.

**Figure 7:**
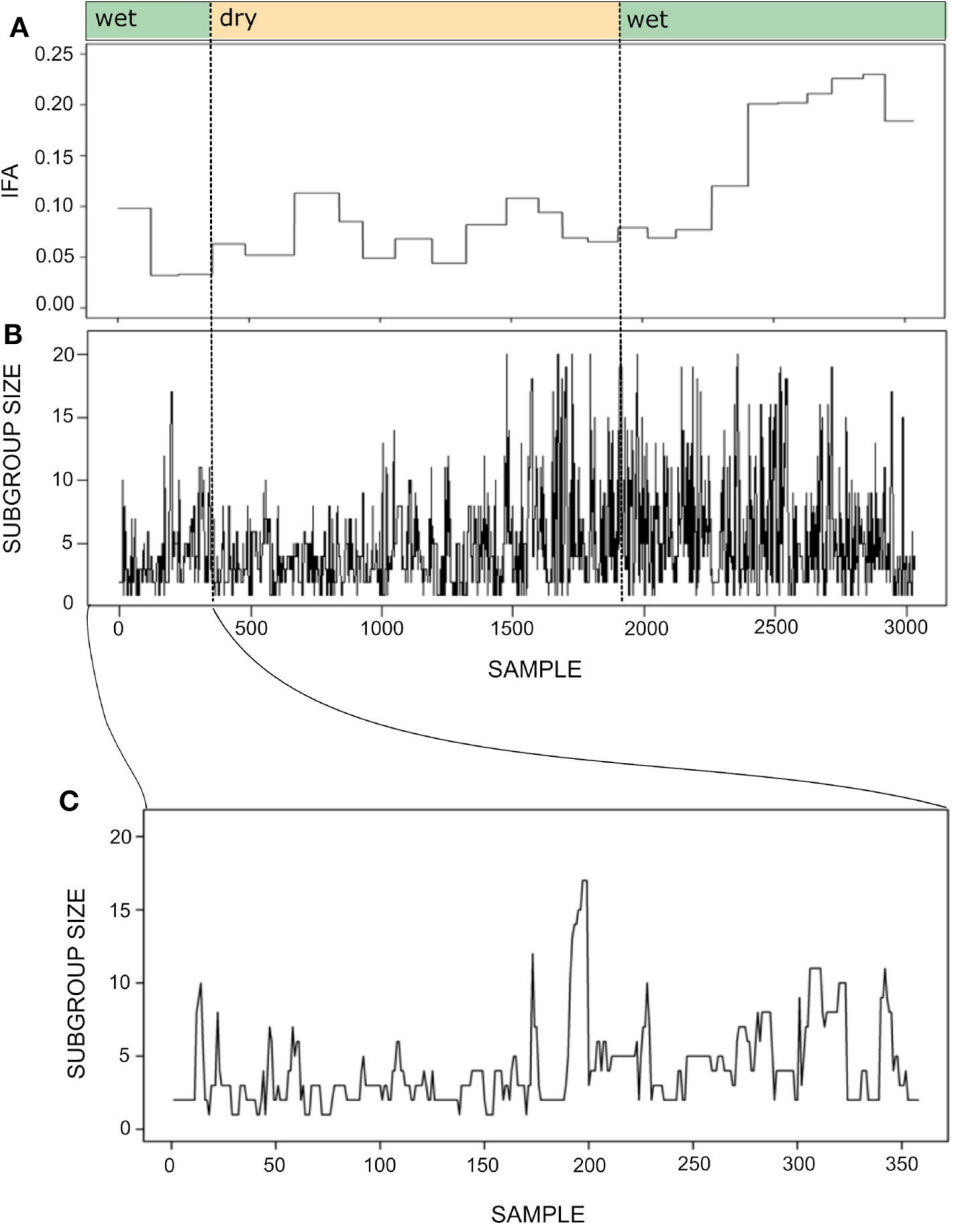
Time series for the index of food abundance (IFA; **A**) and subgroup size **(B)**. The IFA measures the overall abundance of fruit in the spider monkey's habitat, considering their most preferred species, their fruiting status and the abundance and relative size of trees (see section 6). The temporal resolution of the subgroup size data is 20 min, whereas food abundance was monitored biweekly. Thus, the IFA series has the same value throughout a given biweekly period, while subgroup size fluctuates at a much finer temporal scale. Noted above are the seasons (wet or dry) to which each sample belongs. Panel **(C)** presents a fragment of the subgroup size time series showing its variation between September 30 and October 31st 2013. Note that the time series was constructed with sets of scan samples taken every 20' collected throughout 4–8 h periods and that subgroups followed in consecutive days were not necessarily the same. Therefore, the spikes and drops observed in the curve do not always reflect fission or fusion events.

Figure 7 shows the time series for the IFA and subgroup size during one year. As mentioned above, maintaining the temporal resolution of the subgroup size time series was important in order to maintain a sufficient number of continuous series of observations. Despite the different temporal resolution of each time series, it seems that subgroup size increases together with IFA during the second wet season.

In previous work, the match between the collective computation output and the environment was evaluated using mutual information (Brush et al., 2018). Here we use transfer entropy:

(6) $$\begin{array}{l} T_{x\to y}\left(t\right)=H\left(y_{t}|y_{t-1}\right)-H\left(y_{t}|y_{t-1},x_{t-1}\right) \end{array}$$

This is a measure of how much uncertainty in a variable *y* is reduced given past states of both *y* and a variable *x* that is assumed to be independent of *y*. This dependence is over and above the uncertainty about *y* reduced by consideration of its own past state. Here transfer entropy is measuring how much subgroup size uncertainty is reduced by considering past states of subgroup size and IFA, conditioned on the uncertainty reduction by the past states of subgroup size alone. Given the difference in time resolution for the two time series (Figure 7), this implies that, within a given bi-weekly period, we are measuring the transfer entropy between a constant value of IFA and varying values of subgroup size. We used the JIDT package (Lizier, 2014) in *R* (R Core Team, 2017) to estimate the transfer entropy between time series, using the Kraskov estimator with the number of closest neighbors *k* = 4. The two observed time series have a *T*_*IFA* → *SGS*_(*t*)=0.036 nats.

**Figure 8:**
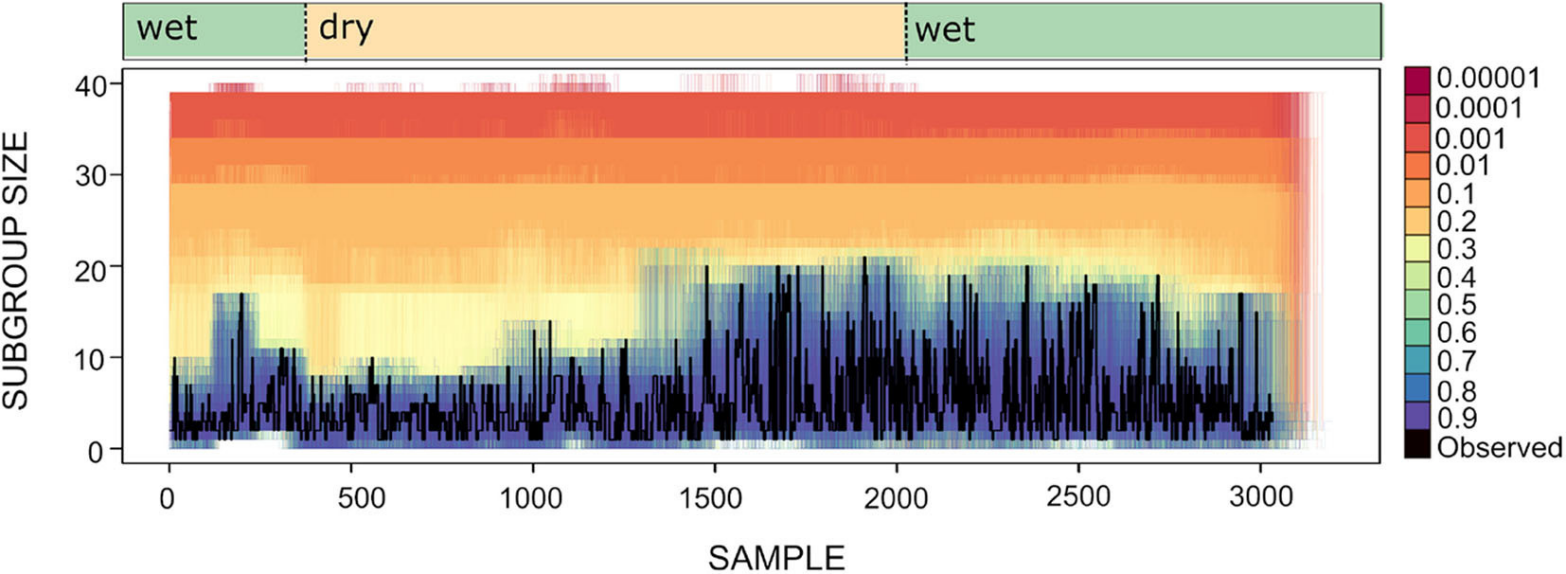
Time series for subgroup size as observed (black line) and simulated (lines of varying color). Each colored line corresponds to an instance of 100 simulations for different values of *U* and *L* = −0.00001. Wet and dry seasons are noted above.

To explore whether spider monkeys collectively compute a subgroup size distribution that is a good match to the distribution of fruiting trees, we assess which of our circuits with different strategy integration rules (described in section 4), computes a distribution of subgroup size that is a good fit to the current abundance of fruiting trees. Shown in Figure 8 is the time series for the subgroup size values together with the subgroup size time series of all simulated data sets generated for different values of *U*. Figure 8 shows what was already apparent in the subgroup size distributions shown in Figure 5, but in the form of a time series: simulated data sets with *U*≥0.4 generate a subgroup size distribution that is closest to the observed distribution.

**Figure 9:**
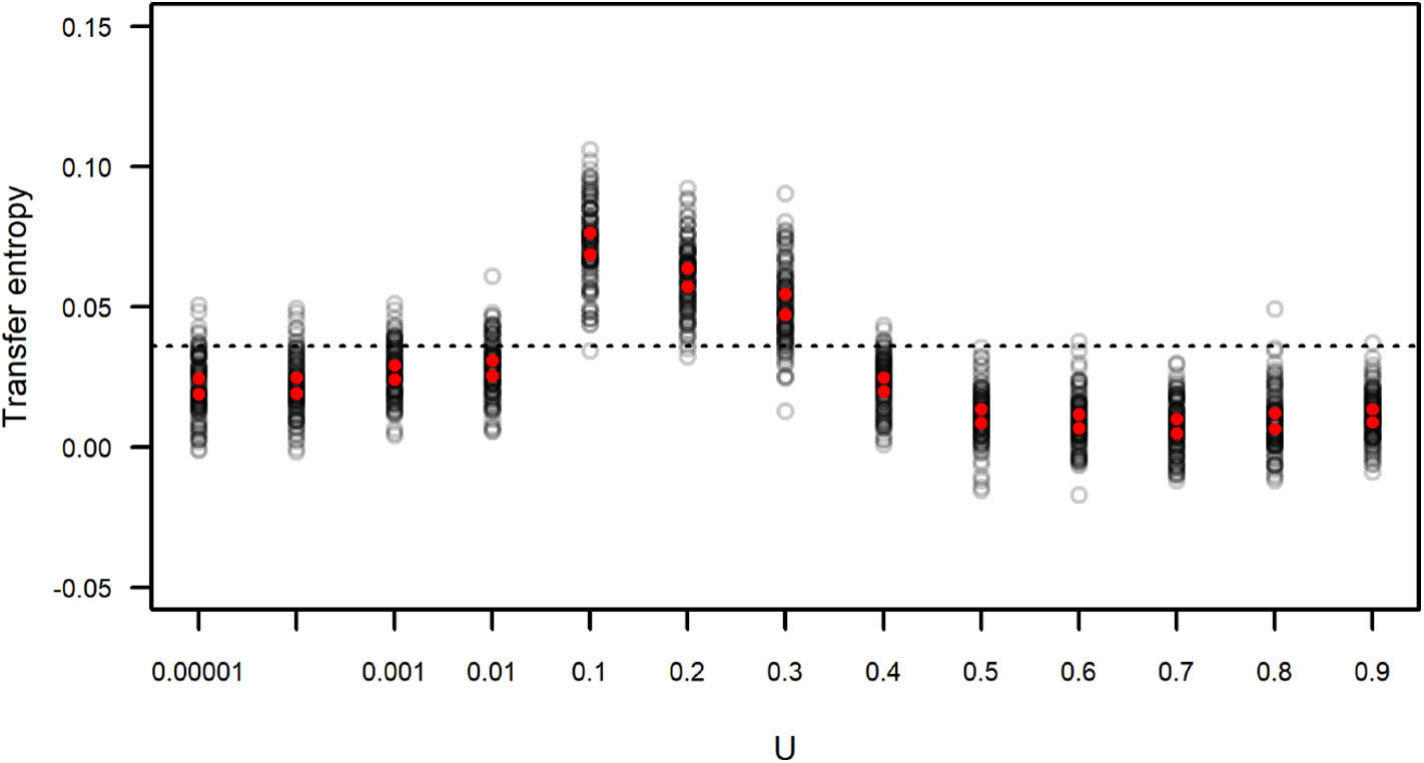
Transfer entropy between simulated IFA and simulated subgroup size. Each gray circle corresponds to an instance of 100 simulations run with varying values of *U* and *L* = −0.00001. Red dots indicate the upper and lower limits of 99 percent confidence intervals of the mean. The dotted line corresponds to the value of transfer entropy found for the observed IFA and subgroup size data in Figure 7.

We calculated the transfer entropy between the IFA time series and its corresponding subgroup size time series. We generated simulated data sets that included the same values of IFA as in the original dataset, but because the observation period length could vary (as the length of each observation period, *n*, was sampled from the distribution of observed *n*) there is a certain degree of variation around the observed data. Each simulated IFA series was compared to its corresponding subgroup size series. These values of *T*_*IFA* → *SGS*_(*t*) are presented in Figure 9, which also shows the value of *T*_*IFA* → *SGS*_(*t*) obtained for the observed IFA and subgroup size time series (Figure 7). The results suggest simulated subgroup size data sets with 0.01 < *U* <0.4 match the temporal variation in IFA values better than the empirically observed subgroup size distribution and better than the simulated distributions computed with *U* ≥ 0.4.

## 7. Discussion

Social structure typically changes slowly compared to the interactions giving rise to it. As such, social structure, whether optimal for the environment or not, reduces uncertainty about the future state of the system and provides a relatively stable background against which individuals can tune their own strategies (Flack, 2017a). Hence there are two challenges for a group computing its social structure: that it changes slowly enough to remain informative for decision-making and that it adaptively tracks the environment.

Frugivorous spider monkeys are faced with two significant sources of uncertainty related to foraging—to discover the location of fruiting trees and to distribute themselves over these fruiting trees to minimize conflict (Aureli et al., 2008) and the costs associated with large groups (Asensio et al., 2009), as well as to maximize resource intake (Symington, 1988). We have used a theory of collective computation (see references in the introduction) to explore how fission-fusion dynamics arises in spider monkey groups and whether the resulting distribution of subgroup size is a good match to the environment. We found spider monkey collectives appear to be able to partially match subgroup size to resource abundance. Our results suggest however that the collective computation of subgroup size is not optimal with respect to food availability as measured by our index.

In simulating the circuits of subgroup-joining strategies we discover values of a sensitivity parameter *U* (a measure of the degree of consensus among the incoming weights required for an individual to make a decision about whether to stay or go) leading to a distribution of subgroup size that is a better match (than the observed distribution of subgroup size) to the observed abundance of fruiting trees. This suggests collective computation is under constraint and the system is experiencing adaptive lag—that is, still learning the best collective strategy to integrate information accumulated by group members. The deviation might instead be spurious–an outcome of (1) the way in which we calculate the food abundance index, (2) the fact that the data used to construct the two distributions are noisy and have different time resolutions: food abundance was measured at a bi-weekly scale while subgroup size was observed every 20 min, or (3) other factors besides social knowledge and relationships contributing to subgroup size decision-making.

We should also be cautious in interpreting the power of the collective computation at small *U* values. In these limits subgroups converge to a constant size where food abundance is expected to be somewhat predictive of size simply because both values remain constant during each bi-weekly period. These caveats aside, whereas collective computation in this system is not optimal, it remains nonetheless predictive and able to capture information about the environment. Specifically, the circuits that capture subgroup joining strategies can aggregate information about the environment. Although we did not study longer timescales, the slowly changing structure of groups provides a means for storing information accumulated by individuals about food availability across years (Palacios-Romo et al., 2019). With individuals that are more than 30 years old (see Supplementary Information), who are using spatial memory for their foraging decisions (Valero and Byrne, 2007), the information made available to the group through their experience is likely an important element to track long-term changes in the foraging environment.

Some means by which computations can be refined maximizing the match between group behavior and the abundance of food, includes individuals changing the way they accumulate information and/or compute strategies for staying or leaving, tuning how individuals integrate over those strategies, and tuning how the strategies interact in the circuit to produce subgroup size distributions. For example, are some individuals' strategies (perhaps because they influence many others) exerting a disproportionate effect on the output or do many individuals contribute in small ways? The problem of how collectives achieve optimal information processing is an important one in biology (Tkačik and Bialek, 2016), and near optimal information processing has been discovered in a number of biological systems (e.g., Petkova et al., 2019). However, these examples tend to be relatively simple developmental mechanisms such as segmentation during development of the fruit fly larval body plan. The circuit approach allows the question of tuning to obtain optimal information processing to be addressed through simulation in more complicated systems.

Additional factors that could affect decision-making, thereby shifting the subgroup distribution from optimal to suboptimal, are a variety of social variables like sex and age, the previous history of interactions, and kinship relationships (Ramos-Fernández et al., 2009; Busia et al., 2017). However, because we are extracting individual strategies directly from the data, these modulating factors are already included in the weights between individuals. Other factors that are currently implicit include the risk of predation or location within the group's home range, which could also affect the subgroup size.

Our results shed light on how a group can best acquire and share information about patchy and dynamic environments. While individual foraging strategies based on spatial knowledge have been well-documented (Janson and Byrne, 2007; Fagan et al., 2013), group foraging strategies are less well-known outside of social insects (Gordon, 2016; cf. Gil et al., 2018). Exchanging information about available patches when foragers disperse and learning about the location and availability of different patches increases the foraging success of the whole group (Falcón-Cortés et al., 2019). The circuit of individual strategies that we infer here is, at least in part, a reflection of information sharing about available patches. Following another individual when ignorant is a simple mechanism of information sharing (Palacios-Romo et al., 2019), that could be reflected in the dyadic weights we have measured. This would lead to a fully connected circuit with information about food sources promoting a flexible grouping pattern that matches heterogeneity in the environment.

It is interesting to compare our approach to that of optimal foraging theory, which would postulate an optimal subgroup size distribution, based on a set of constraints and the best compromise between costs and benefits, which for most cases are unknown (Fretwell and Lucas, 1970; Stephens and Krebs, 1986). An empirical test of this postulate would consist of the match or lack thereof of the observed distribution to the food abundance and this would be interpreted in terms of the unknown mechanisms for how subgroup size comes about (e.g., Chapman et al., 1995). Our approach is more mechanistic: we observe a series of stay-leave decisions resulting from the interactions between individuals and construct a circuit of strategies that serves as a hypothesis for how the subgroup size distribution could emerge. We measure how similar these emerging distributions are to the observed and then test how well the time series matches the environmental variation. That we find alternative circuits that could produce a better match to the environment implies that the system is not necessarily constrained, as would be postulated by optimal foraging theory.

Mutual information, as a measure of uncertainty reduction, has some nice properties. It provides a robust way to study how near optimal a collective behavior is, and this provides a proxy for adaptiveness. We can also study different kinds of uncertainty reduction: an endogenous one, that involves collective computation of social structure that makes the world more predictable for individuals within a system (e.g., Brush et al., 2018); and an exogenous one, whereby collective computation produces social structure that encodes knowledge about resource availability in the environment (this paper). Uncertainty reduction is consistent with a cost-benefit framework without requiring costs and benefits to be estimated. And quantification of the quality of the output of collective computation in information theoretic terms builds a technical bridge to Boltzmann's and von Neumann's ideas about the role of entropy in generating ordered states (Krakauer et al., 2020) that can form the basis of new levels of individuality, even at the social level.

In addition to assessing whether the output matches the environment, we studied the mechanics of collective computation. Previous work suggests spider monkeys preferentially follow food-aware individuals (Palacios-Romo et al., 2019). In the time series we find evidence in support of this result: we are able to extract significant (above-null) pair-wise probabilistic strategies used by individuals to decide to stay in or leave subgroups. Each individual had 20-30 strategies of varying strength (out of 46 possible). Generally the Δ*P* were larger for “stay” strategies than “leave” strategies, suggesting possible food presence is a more important factor to spider monkeys than possible food absence. This emphasis on “attraction” might also be important for maintaining cohesion in fission-fusion dynamics in the context of a heterogeneous foraging environment with multiple alternative foraging options (Ramos-Fernández, 2005; Sueur et al., 2011). The strategies we find also recover well-known social patterns for *Ateles* spp., in particular—same sex based homophily for joining and repulsive tendencies between individuals of different sex (Fedigan and Baxter, 1984; Ramos-Fernández et al., 2009). It remains to be determined whether further, more fine-grained patterns like the frequency of dyadic interactions are also recovered by these strategies.

We used the extracted strategies to construct a family of circuits that vary in how individuals integrate these strategies to produce binary decisions to join or leave a subgroup. Individuals can have both repulsion (leave) and attraction (join) strategies. In previous work (DeDeo et al., 2010), strategies were passed through an AND or OR gate that captured conflict averse (all strategies have to say “go” to join a fight) and conflict prone dispositions (one “go” strategy was sufficient to join). Here we use thresholds. To recover the observed subgroup size distribution in simulation requires sums over strategies (∑Δ*P*≥*U* = 0.4) much larger than the strength of individual strategies (the majority of individual Δ*P* values are below 0.05). This suggests individual-level decisions, as well as the aggregate output, require that individuals take into account relationships and social knowledge of many group members. If so, this would suggest that spider monkeys rely on social information from the wisdom of crowds (e.g., Jayles et al., 2017; Moreno-Gámez et al., 2017; Kao et al., 2018) to make decisions. These decisions are aggregated to collectively compute subgroup size distributions.

Mesoscale strategic circuits are summaries or average tendencies and therefore provide an economical way to process information. Slow variables, encoded in individual strategies, are compressed summaries of noisy interactions (Flack, 2017b). The idea that the mesoscale circuit is a compressed representation of microscopic dynamics has parallels in multiplex networks, which have proven to be a better representation of the dynamics of many systems than the simple aggregation of different layers (De Domenico et al., 2015; Smith-Aguilar et al., 2019). Moreover, this way of compressing information may allow the social structure of spider monkeys to be flexible enough to track a dynamic environment, and, at the same time, be robust to disturbances. This has parallels to neural processing (Bassett et al., 2011; Daniels et al., 2017). As we have discussed elsewhere (see Brush et al., 2013, 2018; Daniels et al., 2017; Flack, 2017a) compression and related principles of collective computation have implications for engineered systems, such as web search and swarm robotics (e.g., Bonabeau et al., 1999; Seth, 2001; Young et al., 2013), as well as pattern recognition by artificial neural networks and human reputation networks.

How spider monkeys collectively compute fission-fusion social structure and how these computations can be tuned to realize adaptive variants raises many questions. Using longer time series, we could ask whether collective computation and fit to the environment are being refined and improved over time. With higher resolution data on strategies, and using methods from information theory (e.g., Rosas et al., 2019), it should be possible to quantify the degree to which the output is irreducibly encoded in the circuit as opposed to decomposeable. Is social knowledge processed in a pairwise manner or do individuals perceive synergistic interactions among group members (e.g., does individual's A perception of individuals B and C contribute non-additively to its social knowledge)?

Understanding how a natural social system carries out adaptive computations could help to improve the performance of artificial systems. For instance, our results could provide insight into the mechanisms underlying learning through backpropagation in artificial neural networks. The way in which individuals adjust their strategic signaling in computing an appropriate power structure that feeds back to provide information about social interaction cost might be analogous to unsupervised learning (i.e., where the target is endogenous to the system) (Flack, 2017a; Brush et al., 2018). A system like the one we study here, with fission-fusion dynamics that can adjust to environmental conditions like the availability of fruiting trees, might be analogous to supervised learning (i.e., where the target is exogenous to the system). In both cases, feedback might share features with backpropagation in the strong and weak senses–the connection weights in the circuits/networks appear to be adjusted with a combination of vector (Brush et al., 2018) and scalar feedback (Flack et al., 2006) to minimize the network's error function when learning a task (Rumelhart et al., 1986; Lillicrap et al., 2020). This is just one of many exciting comparisons that could be made to better understand how different types of feedback, through tuning (Daniels et al., 2017) and downward causation (Flack, 2017a), shape the ability of the circuit to learn. And, as described in the Introduction, collective coarse-graining can produce a coherent mesoscale functioning as an information bottleneck, an ideal that is at least conceptually similar to the information bottleneck described by Tishby and colleagues to explain how deep neural networks encode information parsimoniously (Tishby et al., 2000; Tishby and Zaslavsky, 2015; Flack, 2017a).

We have studied how a natural social system collectively computes. This is achieved through feedback among different scales of social organization, as proposed by Hinde's (1976) early paradigm and made explicit in Flack (2017a) and Flack (2017b). Studying collective computation should also find a range of different applications in the engineering of distributed, adaptive systems (Bonabeau et al., 1999).

## Data Availability Statement

The datasets generated for this study are available on request to the corresponding author.

## Ethics Statement

The animal study was reviewed and approved by the corresponding authorities in Mexico: the Direccion General de Vida Silvestre, Secretaria de Medio Ambiente y Recursos Naturales.

## Author Contributions

All authors conceived the idea for this study. JF and DK developed the theory. GR-F and SS designed the data collection and performed the analysis and simulations. SS collected the data. All authors discussed the results and contributed to the manuscript and giving approval to the final version.

## Conflict of Interest

The authors declare that the research was conducted in the absence of any commercial or financial relationships that could be construed as a potential conflict of interest.
