# Supplementary material for: Collective Computation in Animal Fission-Fusion Dynamics
Source: Front Robot AI. 2020 Jul 21;7:90. doi: 10.3389/frobt.2020.00090 (PMC7805913; doi:10.3389/frobt.2020.00090)
Supplement: Supplementary file 1 [file Data_Sheet_1.PDF]

Supplementary Information for "Collective computation in animal fission-fusion dynamics"

Gabriel Ramos-Fernandez, Sandra E. Smith Aguilar, David C. Krakauer and Jessica C. Flack

1 Details on study site and subjects

The study was based on data collected at the *Otoch Ma'ax yetel Kooh* protected area in Yucatan, Mexico (20°38' N, 87°38' W, 14m AMSL). This area adjoins the village of Punta Laguna and comprises 5376 ha of semi-evergreen forest in patches of vegetation with different successional stages [García-Frapolli et al., 2007]. Yearly precipitation averages ca. 1100 mm with rainfall typically concentrated (70%) between May and October, generating marked seasonal patterns on the vegetation [Smith-Aguilar et al., 2016]. Average monthly temperature averages 24°C but can range from 21°C in January to 27°C in August, which is usually the warmest month of the year, reaching temperatures of 37°C [Sistema Meteorológico Nacional, 2015].

Data were obtained from a habituated group of wild spider monkeys which has been under continuous study since 1997 [Ramos-Fernández, 2005]. All individuals in the group are known and were identified by experienced observers using distinctive markings in their face and/or bodies. The group is normally found within a long-term home range of ca. 44 ha [Smith-Aguilar et al., 2016]. For this study, we considered data from 47 individuals observed between January 2013 and December 2014. These included 25 adults (monkeys $>$ 9 years old; 16 females and 9 males), 11 subadults (5-9 years old; 9 females and 2 males) and 11 juveniles (age 3-5; 5 females and 6 males) totaling 30 females and 17 males. We excluded infants (individuals less than three years old which have no younger siblings and travel mostly carried by their mothers) and any unidentified individuals. As mentioned in the main text, the study period included 5780 scan samples of subgroups collected between January 2013 and December 2014. Within this period, each individual was observed in an average of 663.8 scan samples (range 2 to 2205 scan samples). Therefore, the individual which was observed the most, was present in 38.1% of the total scan samples (Table 1).

References

García-Frapolli, E., Ayala-Orozco, B., Bonilla-Moheno, M., Espadas-Manrique, C., and Ramos-Fernández, G. (2007). Biodiversity conservation, traditional agriculture and ecotourism: Land cover/land use change projections for a natural protected area in the northeastern yucatan peninsula, mexico. *Landscape and Urban Planning*, 83(2-3):137–153.

Ramos-Fernández, G. (2005). Vocal communication in a fission-fusion society: do spider monkeys stay in touch with close associates? *International Journal of Primatology*, 26(5):1077–1092.

Sistema Meteorológico Nacional (2015). Normales climatológicas en la estación 00023012 (Cobá, Quintana Roo), periodo 1980–2010.

Smith-Aguilar, S. E., Ramos-Fernández, G., and Getz, W. M. (2016). Seasonal changes in socio-spatial structure in a group of free-living spider monkeys (ateles geoffroyi). *PloS One*, 11(6):e0157228.

Number	ID	Scan samples present	Scan samples absent	Sex	Age class
1	AE	570	5210	F	A
2	AI	354	5426	M	A
3	AM	838	4942	F	A
4	BE	381	5399	M	A
5	BH	406	5374	F	S
6	BL	16	5764	F	S
7	BO	394	5386	M	A
8	CC	9	5771	M	A
9	CD	3	5777	M	A
10	CH	1862	3918	F	A
11	CT	76	5704	F	S
12	DW	104	5676	M	J
13	EG	1393	4387	M	A
14	EL	9	5771	F	S
15	FD	5	5775	M	A
16	FL	1421	4359	F	A
17	HI	558	5222	F	A
18	JA	1679	4101	F	A
19	JN	1411	4369	M	A
20	KL	1138	4642	F	A
21	KN	17	5763	M	J
22	KO	2205	3575	M	J
23	LB	192	5588	F	J
24	LH	63	5717	F	A
25	LO	1965	3815	F	A
26	LU	2	5778	F	A
27	LX	637	5143	F	S
28	ME	1183	4597	F	S
29	MH	24	5756	M	S
30	ML	324	5456	F	A
31	MO	8	5772	F	A
32	MS	1749	4031	M	S
33	NX	37	5743	F	S
34	NC	6	5774	F	J
35	NN	1575	4205	F	J
36	NK	1446	4334	F	J
37	OC	52	5728	F	A
38	PC	458	5322	F	A
39	SH	8	5772	M	J
40	SK	108	5672	F	S
41	TG	487	5293	F	A
42	TL	1455	4325	M	A
43	VA	1884	3896	F	J
44	VE	1650	4130	F	A
45	VI	269	5511	F	S
46	WB	695	5085	M	J
47	ZO	72	5708	M	J

Table 1: Individuals in the study group, indicating the number of subgroup scan samples in which each individual was observed (from a total of 5780 scans), sex (F= female; M= male), age class (J= juvenile; S=subadult; A= adult).

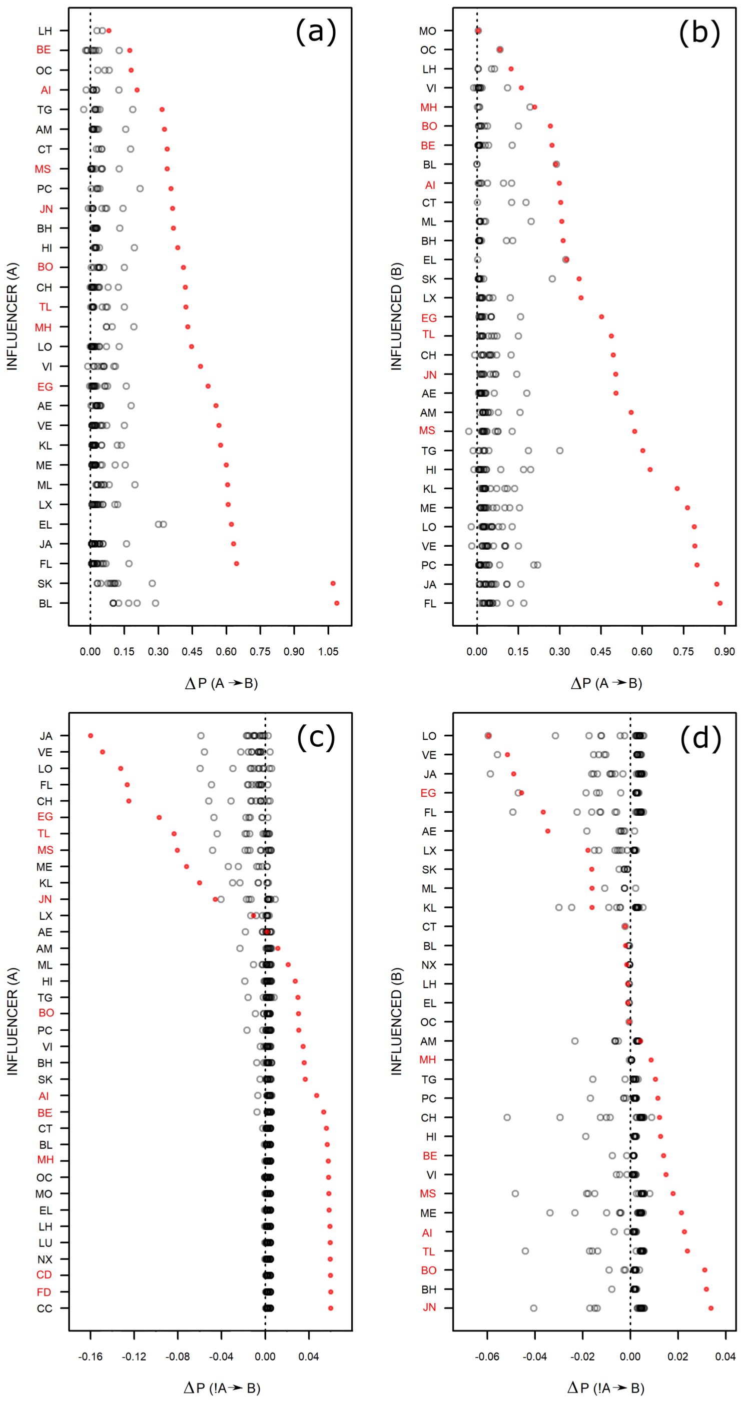

Figure 1: Influence of the presence (a,b) or absence (c,d) of individuals on the presence of group members in a subgroup. Codes in the vertical axes represent individuals, and each circle represents a significant probability (ΔP, see main text for definition) of influencing (a,c) or being influenced (b,d) by another. Because only significant probabilities are shown, the number of circles per individual varies. In (a) and (b), positive values indicate influences which favour the presence of individuals in a subgroup and negative values indicate influences favouring absence in a subgroup. In (c) and (d), positive values indicate that the absence of the influencer favours the subsequent presence in a subgroup of those influenced, while negative values indicate that the absence of the influencer favours the subsequent absence in the subgroup of those influenced. Red points indicate the sum of ΔP values for each individual as influencer (a,c) or influenced (b,d) and are equivalent to the instrength (b,d) or outstrenght (a,c) of the networks depicted in Figure 3 in the main text. Dotted lines are shown as reference to mark zero on the x-axes.
